# Supplementary material for: McKittrick–Wheelock Syndrome, a Rare Cause of Nonresponsive Persistent Dyselectrolytemia
Source: Diagnostics (Basel). 2025 Sep 26;15(19):2459. doi: 10.3390/diagnostics15192459 (PMC12523882; doi:10.3390/diagnostics15192459)
Supplement: Supplementary file 1 [file diagnostics-15-02459-s001.zip › diagnostics-3756631-supplementary.pdf]

Table S1. Biological parameters: hematological and endocrinological parameters, kidney and liver function

Biologic parameter	Determined value	Normal range
Hemogram		
Hemoglobin (g/dl)	15.98	12.6 - 17.4
Leucocytes (cells/µl)	24.36 x10^3	4 - 10
Neutrophils (cells/µl)	22.21 x10^3	2 - 8
Platelets (cells/µl)	375 x10^3	150 - 450
Urea (mg/dl)	209	18 - 55
Creatinin (mg/dl)	3.4	<1.2
eRFG (ml/min/1.73 m^2)	19	>90
ALAT (U/L)	10	<41
ASAT (U/L)	28	<40
ALP (U/L)	94	40 - 150
Total bilirubin (mg/dl)	1.08	≤1
Direct bilirubin (mg/dl)	0.33	<0.2
Glycemia (mg/dl)	136	60 – 99
HbA1c %	6.19	4.0 – 6.0
Urinalysis		
Density	1010	1010 - 1025
Proteins (mg/dl)	25	Negative
Red blood cells (/µl)	neg	Negative
White blood cells (/µl)	neg	Negative
Urine beta-2 microglobulin (mg/l)	0.76	<0.3
Urinary ionogram		
Uric acid (mg/24h)	319	250 - 750
Phosphorus (g/24h)	27.5	0.4 - 1.3
Potassium (mmol/24h)	49	25 - 125
Chloride (mmol/24h)	<20	110 - 250
Sodium (mmol/24h)	<20	40 – 220
Calcium (mg/24h)	<2	100-320
Proteinuria (mg/24h)	1260	0 - 300
Addis-Hamburger test		
Urinary volume/3h (ml)	150	
Urinary output (ml/min)	0.3	
Erythrocytes (no/min)	2490	0 - 1000
Leukocytes (no/min)	19920	0 - 2000

ALAT= alaninaminotransferase, ASAT= aspartataminotransferase, eRFG = estimated glomerular filtration rate, ALP = alkaline phosphatase, GGT = gamma-glutamyl transferase, TSH = thyroid stimulating hormone, FT4 = Free T4, FT3 = Free T3

Table S2. Acid-base and electElectrolyte balance, immunological, inflammation and tumoral status

Biologic parameter	Determined value	Normal range
Acid-base and electrolyte balance		
Serum bicarbonate (mEq/l)	34	22 - 29
Sodium (mEq/l)	125	135 - 145
Potasium (mEq/l)	2.3	3.5 - 5.1
Ph	77	98 – 107
Chloride (mEq/l)	2.37	1.6 – 2.6
Magnesium (mg/dl)	7.5	3.9-5.6
Glucose (mmol/l)	34.79	2.5-7.8
Urea (mmol/l)	263	280 – 295
Serum osmolarity (mOsm/l)	332	50 - 1200
Urinary osmolality (mOsm/kg)		
ESR (mm/h)	58	1 - 10
Ferritin (ng/ml)	550	30 - 400
LDH (U/l)	574	135-225
C-reactive protein (mg/dl)	40.91	<0.5
Procalcitonin (ng/ml)	0.12	<0.5
Serum aldosterone (ng/ml)		
Supine (morning)	72.2	1.76 – 23.3
Copeptin (Provasopresin)(pmol/l)	18	1.0-24.5
Serum protein electrophoresis		
Albumin (%)	0.60	54.3 – 68.0
Alpha 1-globulin (%)	002.9	1.2 – 3.3
Alpha 2-globulin (%)	015.0	6.6 – 13.5
Beta 1-globulin (%)	007.2	6.0 – 9.1
Beta 2-globulin (%)	004.9	2.3 – 5.7
Gamma-globulin (%)	009.1	7.1 – 19.5
Total protein (g/dl)	6.7	6.4 – 8.3

ESR=Erythrocyte sedimentation rate, LDH=Lactate dehydrogenase test, Ig=immunoglobulin
